# Assessment of Impact of Human Leukocyte Antigen-Type and Cytokine-Type Responses on Outcomes after Targeted Therapy Currently Used to Treat Chronic Lymphocytic Leukemia

**DOI:** 10.3390/jcm12072731

**Published:** 2023-04-06

**Authors:** Mihaela Andreescu, Nicoleta Berbec, Alina Daniela Tanase

**Affiliations:** 1 Department of Clinical Sciences, Hematology, Faculty of Medicine, Titu Maiorescu University of Bucharest, 040051 Bucharest, Romania; 2 Department of Hematology, Colentina Clinical Hospital, 020125 Bucharest, Romania; 3 Department of Hematology, Coltea Clinical Hospital, 020125 Bucharest, Romania; 4 Faculty of Medicine, Carol Davila University of Bucharest, 040051 Bucharest, Romania; 5 Department of Hematology, Fundeni Clinical Hospital, 020125 Bucharest, Romania

**Keywords:** chronic lymphocytic leukemia, BTK inhibitors, tumor, treatment strategy, natural killer cells

## Abstract

Tumor growth and metastasis are reliant on intricate interactions between the host immune system and various counter-regulatory immune escape mechanisms employed by the tumor. Tumors can resist immune surveillance by modifying the expression of human leukocyte antigen (HLA) molecules, which results in the impaired presentation of tumor-associated antigens, subsequently evading detection and destruction by the immune system. The management of chronic lymphocytic leukemia (CLL) is based on symptom severity and includes various types of targeted therapies, including rituximab, obinutuzumab, ibrutinib, acalabrutinib, zanubrutinib, idelalisib, and venetoclax. These therapies rely on the recognition of specific peptides presented by HLAs on the surface of tumor cells by T cells, leading to an immune response. HLA class I molecules are found in most human cell types and interact with T-cell receptors (TCRs) to activate T cells, which play a vital role in inducing adaptive immune responses. However, tumor cells may evade T-cell attack by downregulating HLA expression, limiting the efficacy of HLA-dependent immunotherapy. The prognosis of CLL largely depends on the presence or absence of genetic abnormalities, such as del(17p), TP53 point mutations, and IGHV somatic hypermutation status. These oral targeted therapies alone or in combination with anti-CD20 antibodies have replaced chemoimmunotherapy as the primary treatment for CLL. In this review, we summarize the current clinical evidence on the impact of HLA- and cytokine-type responses on outcomes after targeted therapies currently used to treat CLL.

## 1. Introduction

Tumor progression is a complex process that is regulated by a dynamic interplay between tumor cells and the host immune system [1]. Due to the selective pressure employed by the host immune system, tumor cells develop evading mechanisms to avoid detection and consequently elimination [2,3,4]. The host immune system relies on the recognition of antigens presented by human leukocyte antigen (HLA) class I molecules to monitor tumor cells. This function is predominantly facilitated by cytotoxic T cells (CTLs) via their T-cell receptors and by natural killer (NK) cells via their killer-cell immunoglobulin-like receptors (KIRs) [5]. The immune system has the capability to identify and destroy cancer cells by detecting and attacking tumor-associated antigens displayed on the surface of cancer cells. However, certain tumors can evade immune surveillance and persist by developing mechanisms of resistance. One such mechanism is the alteration in HLA expression, which can impair the presentation of tumor-associated antigens to immune cells [6]. 

The mechanism of resistance has been reported in several malignancies, including acute myeloid leukemia and B-cell chronic lymphocytic leukemia (CLL). CLL is characterized by the accumulation of neoplastic B lymphocytes expressing CD19+/CD5+/CD23+ markers in peripheral blood, bone marrow, and lymphoid tissues [7]. Prognostic factors such as clinical staging systems, blood lymphocyte doubling time, serum markers, IgVH mutational status, cytogenetics, and the expression of certain proteins can predict disease progression and outcome [8,9]. The immune system is equipped with robust surveillance mechanisms that identify and eliminate malignant cells, forming the foundation for cancer immunotherapy. By augmenting these immune responses, the goal of cancer immunotherapy is to halt cancer progression [10]. Deciphering the evolutionary trajectory of cancer cells under therapeutic stress is a pivotal undertaking for comprehending the underlying mechanisms that facilitate the development of treatment resistance. This review aims to summarize the current knowledge of the impact of human leukocyte antigen-type and cytokine-type responses on outcomes after targeted therapy currently used to treat CLL.

## 2. Role of Cytokines in Chronic Lymphocytic Leukemia

The interplay between the altered functions of innate and adaptive immune factors is critical in the development, progression, and response to therapy in CLL (Table 1). T cells play a crucial role in the pathophysiology of CLL, and their dysregulation is associated with disease progression [11]. Initially, T cells may exhibit anti-tumor activity, particularly Th1 cells producing interferon gamma (IFN-γ), and influence disease development. Regulatory T (Treg) cells play a crucial role in tumor pathogenesis and the immune surveillance of tumors. Their increased numbers in CLL patients promote a favorable microenvironment for tumor proliferation while suppressing the immune response [12]. T cells in CLL patients exhibit signs of exhaustion, which consequently changes their proliferation and activity [12].

However, the immune alterations manifested in CLL patients are multifaceted. CLL cells are capable of producing almost all cytokines, and changes in the balance of these cytokines can facilitate the growth of the leukemic clone, impacting the survival of CLL cells in vivo by promoting their proliferation and resistance to programmed cell death [13].

Accessory cells such as T cells, bone marrow stromal cells, and dendritic cells produce several factors that function as pro-survival elements in CLL by inhibiting spontaneous programmed cell death [14]. Alterations in lymphocyte and accessory cell subsets along with variations in cytokine expression can contribute to infectious complications, which are a common cause of death in patients with CLL [15]. Furthermore, patients with progressive CLL exhibit a higher number of specific T cells that produce certain cytokines than those with a mild course of the disease. Thus, a modified lymphocyte balance and a distinct cytokine signature can influence the progression of CLL.

There are several aberrant signaling pathways involving various cytokines, including interleukin 2 (IL-2), interleukin 4 (IL-4), interleukin 6 (IL-6), interleukin 8 (IL-8), interleukin 9 (IL-9), and interleukin 10 (IL-10), in CLL. IL-2 is a small cytokine produced by CD4+ T cells in response to antigenic stimuli that activates signal transduction pathways by binding to the IL-2 receptor (IL-2R) composed of IL-2Rα (CD25), IL-2Rβ (CD122), and IL-2Rγ (CD132) subunits [16]. CD25 is essential for the growth and differentiation of immunosuppressive Treg and NK cells. CLL patients exhibit increased concentrations of soluble CD8 (sCD8) and soluble IL-2R (sIL-2R), particularly those with aggressive leukemia, which are related to the disease stage and lymphocyte count [17]. Ig-like transcript 2 (ILT2) is an inhibitory receptor that is significantly decreased on leukemic cells and increased on CD8+ and CD4+ T cells in CLL patients with chromosome 11q deletion. ILT2 has been shown to have immunosuppressive effects by downregulating the growth of both CD4+ and CD8+ T cells, as well as reducing the production and delivery of interleukin 2 by CD4+ T cells. However, an ILT2 block restores cytokine production and T-cell growth [18].

IL-4 is a cytokine that plays a significant role in promoting the differentiation of T cells into Th2 cells as well as in increasing B-cell and T-cell growth, plasma cell maturation, and MHC class II production [19,20]. In CLL cells, there is notable upregulation of IL-4 gene expression, which leads to the activation of an autocrine pathway that shields against programmed cell death or cellular death after DNA damage. This cytokine enhances the survival of CLL cells by stimulating STAT proteins, which activate transcriptional elements involved in cell survival [21]. Moreover, there is an association between miRNAs, such as miR-21-5p and other miRNAs, in the CLCN5 gene and cytoprotection by IL-4 in CLL, implying their potential role in inhibiting apoptosis [22].

In a study by Ruiz-Lafuente et al., the microRNA (miRNA) profile of CLL patients and its response to IL-4 was investigated [23]. The results showed that there were 129 mature miRNAs and 41 of them were differentially expressed compared with normal B cells. Stimulation with IL-4 resulted in an increase in the expression of mature variants of miR-21, which is located adjacent to the VMP1 gene, and miR-362, miR-500a, miR-502, and miR-532 genes, which are mapped within the third intron of the CLCN5 gene. This suggests that the IL-4 signaling pathway may regulate the expression of these miRNAs, which are involved in cellular processes such as gene expression regulation and signal transduction [23].

The CD40L/IL-4 signaling pathway is another pathway that influences CLL, increasing protein synthesis in CLL cells and controlling the translation of DNA damage repair genes, including the ATM gene, which has been linked to overall survival in CLL patients [24]. In CLL cells, the constitutive activation of STAT-3 and NF-κB causes the delivery of IL-6, which further increases JAK2/STAT3 stimulation, leading to the IL-6/JAK/STAT3 feed-forward loop that controls tumor proliferation [25]. The cytokines IL-6 and IL-8 are implicated in the survival and chemoresistance of CLL cells; however, their exact mechanism and source of production are not fully understood. However, an ILT2 block restores cytokine production and T-cell growth, which may provide a promising therapeutic strategy for CLL [26].

IL-9 belongs to the γ-chain cytokine family and binds to both the c-chain receptor and the cytokine-specific IL-9 receptor (IL-9R) [27]. Research has revealed that IL-9 plays a role in neoplastic proliferation, particularly in lymphomagenesis [13]. Studies examined biopsies and serum samples from patients with B-cell chronic lymphocytic leukemia (CLL) and found that the levels of IL-9 mRNA and protein expression were altered and correlated with Rai staging, ZAP70, and CD38 [28]. This was confirmed in further studies, where IL-9 expression was detected in the serum of 20 out of 47 CLL patients but was not detectable in control subjects [29]. Additionally, IL-9 levels were found to be higher in peripheral blood mononuclear cells (PBMCs) from CLL patients than in controls and the levels correlated with β2 microglobulin expression and immunoglobulin heavy variable group (IgVH) status. These findings suggest that increased IL-9 levels may contribute to the onset of CLL, and measuring IL-9 levels may be valuable in predicting the disease prognosis [29].

IL-10 is a cytokine produced by helper T 2 cells, monocytes, and macrophages. Its primary function is to negatively regulate macrophage activity by inhibiting several cytokines, including IL-1, IL-6, IL-8, and TNF-α [30]. Despite its involvement in various biological processes, the role of IL-10 in the development and progression of B-cell chronic lymphocytic leukemia (CLL) remains unclear.

Some studies have suggested that IL-10 may enhance DNA production and alter the migratory capacity of CLL cells [31]. Additionally, IL-10 may promote the growth of CLL cells by augmenting high-affinity IL-2 receptors in cooperation with IL-2. However, the impact of IL-10 on programmed cell death in CLL is still under investigation. Some studies have reported reduced IL-10 levels in lymphocyte cultures from CLL patients, while others have found increased serum IL-10 levels in these patients [32]. The latter finding may contribute to the extended lifespan of CLL lymphocytes in vivo, particularly in Rai stage 2 and beyond, where IL-10 has been shown to block programmed cell death [33]. 

A strong association between IL-10 and lymphocytes has been observed in CLL patients undergoing chemotherapy. In patients who achieved remission, the ratio of regulatory T cells (Tregs) to T helper 17 cells (Th17) and the ratio of IL-10 to interleukin-17 (IL-17) decreased compared with pre-treatment levels. However, in patients who did not achieve remission, the number of Th17 cells and IL-17 levels were increased, while Treg numbers and IL-10 serum levels were decreased, resulting in a decreased IL-10/IL-17 ratio in the remission group. This ratio was associated with several prognostic factors for CLL. The Treg/Th17 and IL-10/IL-17 ratios were inversely correlated with CLL progression and were suggested as markers of leukemia outcomes [34].

Numerous studies have attempted to elucidate the mechanisms by which IL-10 may influence the growth of CLL cells [35]. IL-10 is a potent immunosuppressive cytokine, and IL-10-secreting B cells (B10 cells) have been identified as potent immunosuppressive agents that promote leukemia development [36]. In fact, the number of B10 cells was found to be increased in EμTCL1-Tg mice and was associated with TCL1 expression [37].

**Table 1:** Role of different cytokines in CLL and their mechanism of action.

References	Cytokine	Action	Mechanism
Jaffe et al. [38]	IL-9	Stimulates growth and survival of CLL cells	Activates the JAK/STAT pathway and the PI3K/Akt/mTOR signaling pathway
Alhakeem et al. [39]	IL-10	Suppresses anti-tumor immunity	Has been demonstrated to reduce the generation of effector CD4 and CD8 T cells
Chen et al. [40]	TGF-β	Inhibits proliferation and induces apoptosis	Binds to TGF-β receptor on CLL cells, activating Smad proteins to inhibit cell proliferation and induce apoptosis
Coscia et al. [41]	IL-4	Enhances survival and proliferation of CLL cells Reduces apoptosis	Activates the JAK/STAT pathway and increases the expression of anti-apoptotic proteins
Foa et al. [42]	TNF-α	Induces apoptosis	Binds to TNF receptor on CLL cells, activating caspases to induce programmed cell death
Wang et al. [43]	IL-6	Promotes the survival and proliferation of CLL cells	Activates the JAK/STAT pathway and increases the expression of anti-apoptotic proteins
Huang et al. [44]	IL-2	Enhances the function of CLL cells	Promotes the differentiation and proliferation of CLL cells
Jadidi-Niaragh et al. [45]	IL-17	Promotes the survival and proliferation of CLL cells	Activates the NF-κB pathway and increases the expression of anti-apoptotic proteins
Mo et al. [46]	IFN-γ	Inhibits proliferation	Binds to IFN-γ receptor on CLL cells, activating the JAK-STAT pathway to inhibit cell proliferation
De Cecco [47]	IL-21	Induces the apoptosis of CLL cells	Activates the JAK/STAT pathway and upregulates pro-apoptotic proteins
Gelebart [48]	IL-22	Reduced apoptosis Enhances the survival and proliferation of CLL cells	Activates the JAK/STAT pathway and increases the expression of anti-apoptotic proteins
Cutrona [49]	IL-23	Promotes the survival and proliferation of CLL cells	Activates the JAK/STAT pathway and increases the expression of anti-apoptotic proteins

## 3. Consequence of HLA Class I Antigen Downregulation in CLL

Some studies have shown that CLL cells have lower levels of HLA class I expression than normal T cells from the same individual [50,51]. The findings of these studies have shown that in patients with CLL, there is downregulation of specificities in both HLA-A and HLA-B genes. However, this downregulation is not uniform across all HLA class I alleles, and there are fewer alleles in the HLA-Bw4 group that are affected than those in the HLA-Bw6 group. The HLA-Bw4 group has ligand specificities for inhibitory KIRs, which are important for NK-cell-mediated cytotoxicity [52]. By downregulating HLA class I molecules, CLL cells can evade T-cell-mediated immune responses while increasing their susceptibility to NK-cell-mediated cytotoxicity. This is because the “missing self” inhibitory signal is removed, allowing NK cells to target and kill CLL cells. However, if HLA-Bw4 epitopes are conserved in CLL cells, the “missing self” inhibitory signal is maintained, which theoretically establishes NK-cell tolerance towards these cells [26]. 

Verheyden et al. conducted a study that revealed the downregulation of HLA-A, -B, and -C alleles in CLL cells [51]. Moreover, several studies have indicated that there is a defective cytolytic function of NK cells in CLL, although no differences in natural cytotoxicity receptor expression were observed between CLL patients and healthy age-matched individuals [53,54]. Nevertheless, a clinical data analysis revealed that reduced natural cytotoxicity receptor expression was linked to specific factors, including low hemoglobin levels and elevated lymphocyte counts [55].

## 4. Immune Evasion through HLA Loss of Heterozygosity

Cancer cells often evade the immune system by altering their presentation of neoantigens. One way in which they achieve this is by reducing or eliminating the presence of HLAs on their surface [56]. However, accurately analyzing the number of HLA copies is challenging due to the genetic variability of the HLA locus. The HLA complex is located on chromosome 6 at position 6p21.3. Loss or downregulation of HLA class I expression is a common mechanism by which cancer cells evade the immune system [57]. Loss of heterozygosity (LOH) is the most frequently observed mechanism of HLA haplotype absence in cancer, and LOH at 6p21 is commonly observed in many types of cancer [58]. A study by Drenou et al. reported that in 9 out of 14 cases of non-Hodgkin’s lymphoma, loss of heterozygosity occurred due to the deletion of one allele [59]. Similarly, another study by Lobashevsky reported that pretransplant HLA typing in acute myeloid leukemia patients showed LOH at the HLA gene locus [60]. There are multiple mechanisms that can lead to the reduction or loss of HLA protein expression, which is often associated with gene alterations within the HLA gene cluster. These alterations can affect various genes, including TAP1, TAP2, LMP2, LMP7, and tapasin, as well as the 2-microglobulin locus located at 15q21. One of the most frequent causes of HLA haplotype loss in human tumors is chromosomal loss [61,62]. 

However, aberrations involving chromosome 6p or 15q are not commonly observed in CLL. Nonetheless, Crowther-Swanepoel and colleagues have reported indications of genetic susceptibility and risk loci at 15q21.3 in CLL [63]. Meanwhile, Shah et al. have shown that CLL patients in advanced stages who underwent transplantation exhibited a proclivity towards homozygosity at the HLA-A, HLA-B, and HLA-DRB1 loci [64]. In their 249 study subjects, it was observed that those individuals who were homozygous at one or more loci had a median progression-free survival (PFS) of 25.7 months, whereas those who were HLA-heterozygotes had a PFS of 31.8 months (*p* = 0.007) [64]. These findings suggest that homozygosity that restricts antigen presentation diversity and not necessarily in the context of loss of heterozygosity provides an advantage to tumors in the immune escape process. 

## 5. Human Leukocyte Antigen-G (HLA-G) Role in Immune Evasion

HLA-G is a non-classical HLA class I molecule that differs significantly from classical HLA class I molecules with regards to its genetic diversity, expression, structure, and function [65]. HLA-G exhibits limited polymorphism and is physiologically expressed in specific tissues, such as the trophoblast, thymus, cornea, erythroid, and endothelial precursors. However, in various malignancies, HLA-G expression is abnormally upregulated, which leads to tumor immune evasion, dissemination, and unfavorable clinical outcomes [66]. HLA-G contributes to maternofetal tolerance by acting as a ligand for inhibitory receptors present on uterine NK cells. Additionally, its expression can be induced in different tissues in pathological settings, such as cancer and viral infections [67].

HLA-G is capable of producing seven different variations with a process called alternative splicing. Four of these variations (HLA-G1 to HLA-G4) are attached to the cell membrane, while the remaining three (HLA-G5 to HLA-G7) are soluble [68]. Recent research has explored the potential of HLA-G expression as a prognostic marker in CLL [69,70]. The presence of HLA-G on the surface of CLL cells as measured with flow cytometry has been suggested to have prognostic value. HLA-G expression on CLL cells has been identified as a valuable prognostic factor, as it is associated with disease progression, reduced treatment-free survival, and poor overall survival [69].

The observed prognostic effect of HLA-G expression may be attributed to its tolerogenic properties, which could provide a potential mechanism for evading immunosurveillance and promoting tumor progression. HLA-G exerts various inhibitory functions by directly binding to inhibitory receptors on NK cells, T lymphocytes, and antigen-presenting cells [71]. Despite the direct interaction between immune receptors and tumor cells expressing HLA-G, this may not be adequate to account for the evasion of malignant cells from the immune system. Numerous studies have demonstrated that HLA-G is expressed to varying degrees in most human tumors [72,73].

### HLA-G and Tumor Immunology

HLA-G expression has been found to have diverse effects on malignancies, which include inhibiting the immune system’s ability to kill cancer cells, inducing immune cell death, and promoting the production of immune cells that regulate the immune response. HLA-G can also hinder the movement of different immune cells to the site of cancer growth, either by binding to or transferring receptors from the surface of the immune cells [74,75]. Various receptors that bind to HLA-G have been identified, such as ILT2/CD85j, ILT4/CD85d, KIR2DL4/CD158d, CD8, and CD160, which are present on various cell types [76]. The HLA-G-mediated inhibition of the functions of both innate and adaptive immune cells is dependent on the types of cells and the receptors they express. By binding to receptors on these cells, HLA-G can directly inhibit the functions of natural killer cells, cytotoxic T lymphocytes, B cells, neutrophils, and dendritic cells. Additionally, HLA-G can indirectly promote immune tolerance by enhancing the generation of cells that suppress immune responses, such as T regulatory cells expressing HLA-G, CD4low and CD8low suppressor T cells, Tr1 cells, myeloid-derived suppressor cells, and DC-10 cells. HLA-G can also hinder the movement of immune cells towards the cancer site, which decreases the effectiveness of the immune response against the tumor [77].

HLA-G is known to induce direct immunosuppression by inhibiting the lysis of CTL and NK cells, suppressing allogeneic CD4+ T-cell proliferation and inducing the apoptosis of activated CD8+ T cells and CD8+ NK cells [67]. Additionally, the soluble form of HLA-G (sHLA-G) inhibits B-cell proliferation, differentiation, and immunoglobulin secretion through its interaction with the ILT2 receptor [78]. HLA-G5 can also inhibit the phagocytosis and production of reactive oxygen species by neutrophils through its interaction with ILT4 [79]. HLA-G can also indirectly induce immune tolerance by promoting the generation of various tolerant cells, such as Tregs, DCs, and MDSCs. Trogocytosis is another process with which HLA-G can temporarily inhibit immune responses, as activated immune cells acquire HLA-G-containing membranes from nearby cells [80]. HLA-G-induced Treg cells were observed after allogeneic stimulation with HLA-G1-expressing antigen-presenting cells (APCs), which can result in CD4+ T-cell anergy and the differentiation of suppressive cells. Tolerogenic DCs can also be generated with HLA-G1 tetramers or HLA-G5 dimers via the ILT4-mediated IL-6 signaling pathway and STAT3 activation, ultimately leading to the generation of regulatory T cells [81].

A novel population of tolerogenic dendritic cells (DC-10s) that secretes high levels of interleukin-10 (IL-10) and expresses high levels of the non-classical major histocompatibility complex (MHC) molecule HLA-G and its receptors ILT2, ILT4, and ILT3 has been identified. DC-10s have a specific function in inducing IL-10-producing adaptive regulatory T cells (Tr1) via the ILT4/HLA-G signaling pathway [82]. 

## 6. Targeted Therapies

The therapeutic landscape for CLL is diverse and encompasses multiple therapeutic options, including chemotherapy, immunotherapy, radiation therapy, and stem-cell transplantation. Recently, there have been significant advancements in treatment options, such as targeted therapies that selectively target molecular components involved in tumorigenesis. There are several FDA-approved targeted therapies for CLL, including rituximab, ibrutinib, idelalisib, venetoclax, and acalabrutinib. Despite their high efficacy, the complex biology of CLL allows malignant cells to develop resistance mechanisms to these targeted therapies. 

### 6.1. Rituximab

Rituximab is a chimeric monoclonal antibody that specifically targets the CD20 antigen present on lymphocytes. It induces lymphocyte lysis with antibody-dependent cellular cytotoxicity (ADCC) and complement-dependent cytotoxicity (CDC) [83]. This therapy is widely used to treat various disorders, particularly malignancies such as non-Hodgkin’s lymphomas (NHLs) and CLL [84,85]. Despite its effectiveness, the mechanisms of resistance to rituximab are not entirely clear, as its efficacy depends on the host immune response, which can be influenced by host factors [86]. However, three primary mechanisms of resistance have been proposed. The first mechanism is that tumor cells may have developed ways to block CDC by expressing high levels of membrane complement regulatory proteins (mCRPs), such as CD46, CD55, and CD59. These proteins inhibit the activation of the complement cascade, thereby reducing the effectiveness of rituximab [87]. The second mechanism involves prolonged exposure to rituximab, leading to the downregulation of pro-apoptotic proteins Bcl-2 antagonist/killer (BAK) and Bcl-2 associated X (BAX), which can result in resistance to the therapy [88]. Finally, the most widely supported mechanism of resistance is the downregulation of the target antigen CD20. Studies have identified C-terminal deletions in the CD20 gene and decreased expression of CD20 mRNA in cells that become CD20-negative after rituximab exposure [89,90]. Rituximab is used in different phases of lymphoma treatment, including first-line, maintenance, and salvage phases, and ongoing research aims to develop strategies to overcome these mechanisms of resistance. Preclinical studies have demonstrated that neutralizing mCRPs with antibodies enhances the effectiveness of rituximab, indicating a potential therapeutic approach [91].

In CLL, the ratio of NK cells to malignant B cells affects the ability of NK cells to destroy cancer cells with antibody-dependent cellular cytotoxicity. Therefore, therapeutic interventions that enhance NK-cell activity may be beneficial for CLL treatment. Laprevotte et al. conducted a study to investigate the effects of recombinant human interleukin-15 (rhIL-15) on autologous NK cells in CLL samples [92]. The researchers demonstrated that rhIL-15 stimulated and expanded NK cells, leading to a reduction in malignant B cells. This effect was further enhanced in the presence of an anti-CD20 monoclonal antibody. Moreover, the study revealed a synergistic effect of promoting NK-cell growth exerted by rhIL-15 signaling and CD16 signaling, as obinutuzumab was found to be more effective than rituximab. The growth of NK cells in response to rhIL-15 was dependent on their contact with CLL cells, which was identified as an additional factor facilitating rhIL-15 transpresentation. These findings suggest that rhIL-15 can initiate NK-cell-mediated immunotherapy in CLL, highlighting the importance of NK-cell-mediated cytotoxicity in the treatment of this disease [92].

Furthermore, the administration of rhIL-15 may potentially reduce the immunosuppressive effects of transforming growth factor-beta (TGF-beta) in CLL models, leading to an increase in rituximab (RTX)-mediated antibody-dependent cell cytotoxicity (ADCC) [93]. Elevated levels of TGF-beta were observed in both in vivo and in vitro CLL samples, indicating its involvement in immune dysregulation in CLL [94]. Additionally, TGF-beta can inhibit the production of interferon gamma (IFN-gamma) mediated by CD16 and ADCC in NK cells in healthy individuals [95]. Khouri et al. conducted a study on post-transplant immunomanipulation with rituximab and donor lymphocyte infusion in 43 patients [96]. The researchers found that patients positive for HLA-A1, and negative for HLA-A2 and HLA-B44 (HLA-A1, non-A2, and non-B44) had a statistically significant improvement in complete remission (CR) rates and progression-free survival (PFS) relative to other HLA types. This combination of HLA allele characteristics was also associated with an improvement in response to immunomanipulation [96].

### 6.2. Ibrutinib

The signaling pathway mediated by the B-cell receptor (BCR) and its constituent, Bruton’s tyrosine kinase (BTK), is implicated in the pathogenesis of various B-cell malignancies, such as CLL, mantle cell lymphoma (MCL), and diffuse large B-cell lymphoma (DLBCL) [97]. The constitutive activation of the B-cell receptor signaling pathway in which BTK plays a crucial role has been associated with various B-cell malignancies. This persistent signaling leads to the activation of oncogenic NF-kB and other signaling pathways that promote the survival and proliferation of malignant B cells, leading to their accumulation in the bone marrow, blood, and secondary lymphoid organs [98,99]. BTK is a significant contributor to the pathogenesis of B-cell lymphomas, as its phosphorylation is considerably increased in malignant B cells compared with normal B cells [100]. Ibrutinib was specifically developed to inhibit BTK and has been approved by the FDA for the treatment of several malignancies, including CLL, MCL, and DLBCL [101]. Woyach investigated the mechanism of resistance to ibrutinib in patients that showed relapse in CLL during the treatment period [102]. Their findings showed that resistance to ibrutinib is caused by mutations in the cysteine residue, where the drug binds. Furthermore, there are two additional mutations in BTK or downstream enzymes in the B-cell signaling pathway, such as PLCG2. Some studies have shown that mutations in BTK and PLCG2 are the most common mechanisms of resistance to ibrutinib in CLL, WM, and marginal zone lymphoma (MZL) [103,104].

A case study by Furman et al. investigated a case of a 49-year-old woman who had a diagnosis of CLL and developed progressive disease after 21 months post-ibrutinib therapy. RNA sequencing revealed a mutation of BTK (C481S) in the patients that was not present before the therapy [105]. Similarly, a study by Amin et al. identified 5 out of 48 CLL samples as more resistant to ibrutinib after relapse after chemotherapy [106]. The findings further revealed that three samples had acquired a del17p/TP53 mutation. The study also showed that CLL samples that had del17p/TP53-mutated cells demonstrated less sensitivity to ibrutinib-induced apoptosis [106]. Another study by Kanagal-Shamanna et al. sequenced mutations in 29 genes associated with CLL that developed resistance to ibrutinib/acalabrutinib [107]. They found mutations in *TP53*, *SF3B1*, and *CARD11* after disease progression [107]. Other mutations associated with ibrutinib include the deletion of the short arm of chromosome 8 (del(8p)), which leads to deficiency in a protein called TRAIL in combination with driver mutations in genes such as *EP300*, *EIF2A*, and *MLL2* [108]. Another mutation associated with ibrutinib resistance is a newly identified mutation in the BTK gene (*BTKT316A*), which activates a protein called PLCG2 in CLL [109]. These findings suggest that multiple genetic factors contribute to the development of ibrutinib resistance in CLL, and identifying these factors could help to develop new strategies for treating drug-resistant diseases.

Ibrutinib has been shown to decrease the expression of CD200 and BTLA molecules that cause immunosuppression in CLL cells [110]. In a recent study conducted by Long et al., the effect of ibrutinib and acalabrutinib therapy on the T-cell phenotype, immune function, and CLL cell immunosuppressive capacity was evaluated [110]. Their findings showed that in patients with CLL, the medication ibrutinib was found to significantly increase the number of CD4+ and CD8+ T cells, particularly in the effector/effector memory subsets. This may have been due to ibrutinib’s inhibition of ITK, which appears to reduce activation-induced cell death. Both medications reduced the expression of PD-1 and CTLA-4 in T cells, which are proteins that suppress immune responses. The number of regulatory T cells (Treg) did not change, but the ratio of Tregs to conventional CD4+ T cells decreased with ibrutinib but not with acalabrutinib. Both medications also reduced the production of immunosuppressive molecules CD200 and BTLA, as well as IL-10, by CLL cells [110].

Manukyan et al. conducted a study to investigate the effects of short-term and long-term ibrutinib treatment on the expression of HLA-DR in CLL cells, T cells, and monocytes [111]. The study involved 16 patients with high-risk CLL who were treated with ibrutinib. The researchers analyzed the immune cells in their blood and observed that HLA-DR expression on CLL cells decreased, while the number of CLL cells increased after commencing ibrutinib treatment. Furthermore, when CLL cells were cultured with ibrutinib in a laboratory, a decrease in HLA-DR expression was observed at both the protein and mRNA levels. However, after one month of treatment, an increase in the number of CD4+ and CD8+ T cells, as well as CD4+ and CD8+ cells expressing HLA-DR, was observed. The decrease in HLA-DR expression on CLL cells was temporary and gradually increased by the 12th month of treatment. Long-term treatment with ibrutinib was found to be associated with an increase in the number of CD4+ cells expressing HLA-DR and an elevation of HLA-DR expression on all monocyte subsets [112].

### 6.3. Idelalisib

The PI3K signaling pathway plays a crucial role in the progression of many cancers, and targeting PI3K has emerged as a promising therapeutic approach [112]. However, the existence of four different PI3K isoforms with partially overlapping functions and varying toxic effects presents a significant challenge. Idelalisib, a selective inhibitor of the delta isoform of PI3K, has demonstrated impressive efficacy in treating B-cell malignancies with acceptable side effects and has been approved by the FDA for CLL, FL, and SLL treatment [113]. In vitro studies on CLL cells have identified that resistance to idelalisib is associated with increased expression of insulin-like growth factor 1 receptor (IGF1R). Furthermore, treatment re-sensitization was achieved with an IGF1R inhibitor [114]. Another study found that CLL cells developed resistance to idelalisib with increased and constitutive MAPK pathway activation, making communication between the PI3K and MAPK pathways that bypassed PI3K inhibition possible [115]. The study also identified that increased MAPK pathway activation was associated with mutations in KRAS, BRAF, and MAP2K1 [115]. Resistance to idelalisib has been primarily studied in solid tumors and has been attributed to alterations that increase the activity of PIK3CA, NRAS, or KRAS [116,117].

### 6.4. Venetoclax

Venetoclax is an FDA-approved drug that inhibits B-cell lymphoma 2 (Bcl2), a pro-survival protein that regulates the intrinsic apoptosis pathway [118,119]. The drug binds to Bcl2, allowing pro-apoptotic proteins such as BIM and BH3 to activate BAX and BAK, leading to apoptosis and inhibiting cell proliferation. The regulation of programmed cell death, or apoptosis, is governed by the intricate interplay between pro-apoptotic and anti-apoptotic members of the BCL2 protein family [120]. Anti-apoptotic proteins such as BCL2, BCL-XL, MCL1, and BCL-w exert their survival-promoting effects by countering the pro-apoptotic signals. The pro-apoptotic members can be classified into two subtypes based on their structure, namely, multidomain proteins, such as BAX and BAK, and BH3-only proteins, such as BID, BIK, NOXA, PUMA, BAD, and BIM. BH3-only proteins activate apoptosis by either inhibiting the anti-apoptotic proteins or directly activating the multidomain pro-apoptotic proteins [121]. Hence, the balance between pro- and anti-apoptotic proteins is a crucial determinant of cellular fate in response to apoptotic stimuli [122]. However, malignant cells can develop resistance to venetoclax with various mechanisms, including mutations in the BH3 binding groove of Bcl2 or mutations in Bcl2 itself [122]. Patients with relapsed or refractory (R/R) CLL may also exhibit genetic aberrations in cancer-related genes that confer resistance to treatment. A study by Herling et al. sequenced eight CLL patients that developed resistance to venetoclax [123]. Their findings showed recurrent mutations in BTG1, CDKN2A/B, BRAF, and CD274 (PD-L1) [124]. To improve the clinical efficacy of venetoclax, combination treatment strategies with other agents, such as cytarabine, ibrutinib, rituximab, or bendamustine, have been developed, resulting in improved response rates. Ongoing studies are being conducted to identify optimal combination regimens for venetoclax [122].

Increased monocyte HLA-DR expression has previously been linked to improved cytokine response. A study by Svanberg et al. investigated monocyte and neutrophil phenotype functions in CLL patients who were treated with 420 mg for 8 weeks followed by venetoclax for 5 weeks [123]. The study involved nine participants, and their monocyte and neutrophil counts, as well as the distribution of mature and immature neutrophils, were initially found to be within the normal range. These measurements remained stable throughout the course of treatment. At baseline, the expression of HLA-DR on monocytes was found to be suppressed but significantly increased after combination treatment with ibrutinib and venetoclax (*p* = 0.04). Furthermore, the HLA-DR expression on neutrophils was initially high and did not change after ibrutinib treatment (n = 8) but declined significantly after the addition of venetoclax (n = 7) (*p* < 0.01). The study also found that the LPS-stimulated production of TNF-α and IL-6 was initially suppressed. However, the IL-6 levels increased significantly upon ibrutinib monotherapy, and the levels of both TNF-α and IL-6 almost returned to normal upon the addition of venetoclax to ibrutinib treatment (*p* = 0.02 for TNF-α and *p* = 0.009 for IL-6) [123].

## 7. Conclusions

The host immune system plays a critical role in shaping the tumor microenvironment and influencing clinical outcomes in cancer patients. The CLL prognosis is assessed using clinical stages and biological markers, but the concept of tumor immune escape suggests that the altered expression of HLA molecules may contribute to disease progression. While there is controversy surrounding the evidence supporting immune escape via altered HLA expression in CLL, studies have linked the downregulation of HLA class I antigens to a poorer prognosis. The relevance of HLA expression abnormalities in this context requires further investigation to determine their potential as prognostic markers and targets for precision therapies. Furthermore, cytokines have emerged as key mediators of the tumor microenvironment in CLL, as they play a significant role in the pathogenesis and progression of the disease. The dysregulated production and signaling of cytokines, including IL-6, TNF-α, and TGF-β, have been implicated in promoting CLL cell survival, proliferation, and immune evasion. Although new BTK inhibitors and cell-based therapies provide hope for patients with CLL, research is necessary to optimize treatment sequencing and address knowledge gaps. Future research exploring HLA expression as a potential prognostic marker and target for precision therapies represents a critical area of investigation for advancing CLL treatment decision making and addressing unmet needs in CLL therapy.

## Data Availability

Not applicable.
